# 

**Published:** 1889-03

**Authors:** 


					﻿1889.
OLD SERIES
VoU XXV
1855 v
'new series j
Vol. VI -No I
S fjDURNAi^^^
II. V. M. AlklMblZI). L'hW JR
^bushed By JAMES P.HARRISON&CC
♦________________«aTLANTA,GA,_____J
SUBSCRIPTION t 12.00 A VEAP.^
				

